# Supplementary material for: Are carbon emissions trading and green financial instruments synergistic? -Comprehensive quantitative research based on content analysis
Source: PLoS One. 2024 Mar 7;19(3):e0298601. doi: 10.1371/journal.pone.0298601 (PMC10919601; doi:10.1371/journal.pone.0298601)
Supplement: S1 Data — (ZIP) [file pone.0298601.s001.zip › Data/地级市Z_F工作报告.zip!/╜¡╬≈/╙Ñ╠╢╩╨/└┤╫╘╣1⁄2╓┌║┼í╛┬φ┐╦╩2╛▌═°í┐_2017.docx]

各位代表：

现在，我代表市人民政府，向大会报告政府工作，请予审议，并请市政协各位委员和列席会议的同志提出意见。

一、过去五年工作回顾二号首长

五年来，在省委、省政府和市委的坚强领导下，全市上下深入贯彻习近平总书记系列重要讲话精神和治国理政新理念新思想新战略，紧紧围绕“主攻项目、决战‘三区’，凸现特色、实现跨越”总体要求，统筹做好稳增长、促改革、调结构、惠民生、防风险各项工作，全力推进“8+1”重点工作，顺利完成“十二五”规划目标任务，实现“十三五”良好开局，连续三年位列全省市县科学发展综合考评二类市第一，在建设富裕秀美宜居和谐鹰潭的生动实践中迈出了新的步伐。

（一）过去的五年，是经济平稳较快增长、综合实力显著增强的五年。2015年，全市实现生产总值639.26亿元，是2011年的1.5倍，年均增长10.3%；财政总收入112.43亿元，是2011年的1.6倍，年均增长12.1%；一般公共预算收入83.01亿元，是2011年的2.1倍，年均增长20.9%；规模以上工业增加值358.61亿元，是2011年的1.6倍，年均增长12%；固定资产投资531.56亿元，是2011年的1.9倍，年均增长17.7％；社会消费品零售总额171.71亿元，是2011年的1.7倍，年均增长13.5%。2016年，预计全年实现生产总值688亿元，财政总收入121.3亿元，固定资产投资611亿元。

（二）过去的五年，是结构调整力度加大、发展方式加快转变的五年。三次产业结构由2011年的8.9：64.6：26.5调整为2015年的7.7：59.4：32.9。工业转型发展。“七位一体”铜产业体系(1)初具雏形，内陆首家铜期货交割库、铜现货市场和中江国际商品交易中心顺利运营，2015年铜精深加工占比较2011年提升25个百分点，规模以上铜企业主营业务收入达3091亿元。水工、节能照明、大健康、眼镜等非铜特色产业产值实现4年翻番，2015年市属工业增加值占规模以上工业增加值比重达61.5%，较2011年提高19个百分点。鹰潭高新区升格为国家高新技术产业开发区，并与贵溪经开区双双跻身全省工业园区十强。我市列入国家工业绿色转型发展试点城市和国家第二批资源综合利用“双百工程”示范基地，高新区列入国家循环化改造示范试点，贵溪市列入国家首批创建循环经济示范县。旅游业集群发展。我市列入国家级旅游业改革创新先行区和国家全域旅游示范区创建单位，龙虎山跻身国家5A级旅游景区。寻梦龙虎山、花语世界、圣井山漂流、逍遥城综合体等精品旅游项目建成运营，成功承办第三届国际道教论坛和全省旅游产业发展大会，连续举办八届“海峡两岸道文化艺术交流论坛”。全市接待国内游客年均增长30.4%，旅游总收入年均增长35.1%。商贸物流业加快发展。汽车、机电五金等一批专业市场和鹰潭电商创新孵化园、贵溪电商生态产业园等一批电商集聚区建成运营。2015年货运业销售收入达60.49亿元，电子商务产业交易额达196亿元。市现代物流园被评为全国优秀物流园区。现代金融业稳步发展。在全省率先完成农信社改制并组建鹰潭农商银行，江西银行、九江银行、上饶银行、兴业银行在鹰设立分支机构，4家企业在“新三板”、5家企业在上海股交中心挂牌，市工业控股有限公司和中小企业信用担保有限公司组建运营。五年来发行企业债券、中期票据等直接融资规模达100亿元。现代农业集约发展。成功创建贵溪国家级现代农业示范区，雷溪、杨溪列入省级现代农业示范区。建立了市县乡村四级土地流转服务体系，土地流转面积36.2万亩、流转率达40.2%。2015年，全市粮食总产达72.99万吨，市级以上农业产业化龙头企业达179家，农民合作社达2007家，家庭农场达420家。

（三）过去的五年，是新型城镇化步伐加快、城乡面貌明显改观的五年。国家新型城镇化综合试点和省级“多规合一”(2)试点建设稳步推进，全市建成区面积由2011年的62.9平方公里拓展到2015年的77.2平方公里，城镇化率达55.78%，较2011年提高6.34个百分点。基本完成市城乡总体规划暨“多规合一”（2015-2030年）和新型城镇化规划（2014-2020年）编制。信江新区30平方公里内的主干路网全面贯通，建成区面积达8平方公里，形成了“一江两岸”城市格局。沪昆高铁鹰潭段、320和206国道升级改造、市民服务中心、高教园区、高铁北站、市民广场、北极阁、信江浮桥等一批重大项目建成启用，累计完成棚户区和城中村改造1.41万户，完成主城区30余条主次干道改造，小街小巷改造惠及群众8万余人。新改建信江两岸十里绿色景观长廊、西湖湿地公园等一批特色公园，信江湿地公园列入国家湿地公园试点，成功创建国家园林城市和江西省文明城市。我市列入国家信息惠民城市和国家智慧城市试点，成为全省首个上线运行“智慧城市服务平台”城市和“全光网城市”。贵溪东城区、余江城东新区、月湖新城建设步伐加快，中心镇标准化和秀美乡村建设全面铺开，新开通城市（际）公交线路11条，新改建农村公路1725.2公里，4个乡镇列入国家重点镇，建成秀美乡村点1151个。

（四）过去的五年，是改革开放纵深推进、创新活力不断增强的五年。重点领域改革稳步推进。综合行政执法体制、贵溪和余江深化县城基础设施投融资体制等10项改革列入国家试点，政府购买公益性岗位等14项改革列入省级试点。政府机构改革和“放管服”改革成效明显，政府部门权责清单公布实施，累计取消行政审批项目115项、下放77项，非行政许可审批事项全部取消。建立了市县乡村四级便民服务体系，联审联批系统上线运行。164家中介服务机构入驻“中介超市”。“12345”政务服务热线运行顺畅。市县两级不动产统一登记中心挂牌成立。农村土地承包经营权确权登记颁证工作基本结束，国有林场改革全面完成，余江国家农村宅基地制度改革试点工作成效明显。全方位开放力度加大。累计引进省外5000万元以上项目实际进资906.77亿元、利用外资8.17亿美元，分别年均增长18.3%、13.3%。关检合作“三个一”(3)改革不断深化，开通宁波至鹰潭转检通道，累计实现外贸出口35.91亿美元、年均增长11.8%，连续七年被评为全国外贸百强城市。创新创业加速推进。创建国家级、省级科技创新平台33个，铜检中心取得国家认证认可资质，高新区获批筹建国家知名品牌创建示范区，连续14年被评为国家科技进步先进市。商事制度改革成效明显，实行“五证合一、一照一码”(4)，累计新登记企业3927户，增长31.25%。

（五）过去的五年，是绿色发展取得实效、生态环境更加宜居的五年。

五年来，空气质量优良率保持在85%以上，主要河流断面水质、城市集中饮用水水源地水质达标率均为100%，“十二五”减排任务全面完成。国家城乡生活垃圾第三方治理试点建设加快推进，城南、信江新区、龙虎山等污水处理厂竣工投运，规模化畜禽养殖场污染整治和标准化改造全面推开，贵冶周边修复治理重金属污染土壤2075亩，打造全省城乡最干净的城市取得明显成效。境内所有河流和水库四级“河长制”组织体系实现全覆盖，全市92%以上水库水质达III类以上标准。累计完成造林绿化27.92万亩，森林覆盖率达58.2%，成功创建国家森林城市，建成阳际峰国家自然保护区，贵溪市列入全国造林绿化先进县（市），余江县列入全省首批生态文明先行示范县。

（六）过去的五年，是民生保障持续改善、人民幸福指数不断提高的五年。连续三年实施十大惠民共享工程，一般公共预算支出中民生支出比重达70%以上。城乡居民人均可支配收入分别年均增长11.4%和13.1%。累计新增城镇就业13.7万人，转移农村劳动力8.41万人，连续5年城镇登记就业率稳定在96.5%以上。社会保障制度实现城镇职工、城乡居民全覆盖，连续8年提高基本医疗保险和大病补充医疗保险待遇水平，连续12年提高退休职工养老金待遇，在全省率先建立“多险合一”(5)社保信息系统，全市3.5万名被征地农民纳入城镇职工养老保险，新型农村和城镇居民社会养老保险工作获国务院表彰。城乡低保标准较2011年分别增长33.3%和45.8%，累计发放城乡低保资金6.3亿元。累计建成保障性住房6.7万套，中心城区保障性住房覆盖率达25%，居全省前列。完成农村危房改造8745户、渔民上岸安居工程177户。全力推进脱贫攻坚，2015年减贫7826人。各类教育均衡发展水平进一步提高，江西师范高等专科学校新校区正式开学，完成市直中职教育“三校合一”整合。国家城市公立医院综合改革试点成效明显，村级卫生计生服务室标准化建设实现市域全覆盖，“阳光医药”工作获国务院充分肯定。我市评为全国国土资源节约集约模范市。全民健身活动蓬勃开展，体育健身场所覆盖100%的社区和62.9%的行政村。创作了《七彩畲乡》、《青山作证》、《道之韵》等一批文艺精品，对台千人专场授箓活动影响深远，大上清宫遗址考古发掘获国家文物局批复。全面二孩政策稳步实施。推动军民融合发展，首次荣获全国双拥模范城。平安鹰潭、法治鹰潭建设扎实推进，食品药品安全、安全生产和治安信访形势持续平稳，“六五”普法规划全面完成，连续3年被评为全省社会治安综合治理目标管理先进市，首次荣获全省“平安杯”。政府廉政建设卓有成效，履行党风廉政建设主体责任意识显著增强，反腐倡廉制度建设不断创新，全面落实中央八项规定精神和国务院“约法三章”及省市各项规定，严肃查处了各类违纪违法案件。认真办复人大代表建议、政协委员提案，代表、委员满意率100%。统计、档案方志、广播电视、外事侨务、气象、防灾减灾、人防民防、妇女儿童、老龄、残疾人等工作取得了新的成绩。

各位代表，五年发展的历程极不平凡，取得的成绩来之不易，积累的经验弥足珍贵。这是省委、省政府和市委坚强领导的结果，是历届班子真抓实干、务实推进的结果，是市人大法律监督、市政协民主监督的结果，是全市人民锐意进取、顽强拼搏的结果。在此，我代表市人民政府，向全市广大干部群众，向各位人大代表、政协委员、各民主党派、工商联、无党派人士和社会团体，向驻鹰单位、人民解放军、武警官兵、公安民警和离退休老同志，向所有关心、支持、参与鹰潭经济社会发展的各界人士，表示衷心的感谢，并致以崇高的敬意！

在看到成绩的同时，我们也清醒地认识到，我市经济社会发展还存在不少矛盾和问题，主要表现在：经济总量偏小，城市人气不旺；产业结构不够合理，转型升级任务艰巨；城乡基础设施和公共服务配套还不完善，城市管理和社会治理水平有待加强；创新意识和创新能力不足，人才资源较为缺乏；发展环境仍有待加强，服务型政府建设还需进一步加快。对这些困难和问题，我们一定高度重视，采取有效措施，认真加以改进和解决。

二、今后五年工作的总体要求和奋斗目标

今后五年，是我市同步全面建成小康社会的决战期、实施“十三五”规划的重要期和推进“四个鹰潭”建设的关键期。市第八次党代会描绘了今后五年鹰潭发展的宏伟蓝图。立足新起点，必须把握新要求、担当新使命、实现新作为。

今后五年政府工作的总体要求是：高举中国特色社会主义伟大旗帜，以马克思列宁主义、毛泽东思想、邓小平理论、“三个代表”重要思想、科学发展观为指导，全面贯彻党的十八大和十八届三中、四中、五中、六中全会精神，深入贯彻习近平总书记系列重要讲话精神和治国理政新理念新思想新战略，统筹推进“五位一体”总体布局和协调推进“四个全面”战略布局，牢固树立五大发展理念，紧紧围绕省委、省政府决策部署和市第八次党代会精神，突出“全域一体”，做好铜产业、旅游业、物流业“三篇文章”，更加注重改革开放，更加注重创新创业，更加注重城乡统筹，更加注重绿色发展，更加注重民生保障，不断提升世界铜都首位度、中华道都知名度、田园新城宜居度，为建设富裕秀美宜居和谐鹰潭、同步全面建成小康社会而不懈努力。

今后五年我市经济社会发展的主要奋斗目标是：

——经济综合实力进一步增强。到2021年，生产总值突破1000亿元，财政总收入突破200亿元，规模以上工业增加值突破500亿元，固定资产投资突破1000亿元，社会消费品零售总额突破300亿元。

——产业转型升级进一步加速。深入实施创新驱动发展战略，三次产业结构调整为5:55:40，市属工业增加值占规模以上工业增加值的比重达66%，铜精深加工、铜基新材料产品产值占比80%以上，非铜特色产业主营业务收入突破1000亿元，高新技术产业产值年均增长12%以上，科技进步贡献率达60%以上。大众创业、万众创新蓬勃发展，非公有制经济健康发展。

——城乡统筹步伐进一步加快。市域空间发展格局进一步优化，“多规合一”深入实施，城乡公共资源配置更为均衡，城镇化率达63%。中心城区集聚和辐射能力显著增强，市本级持续快速发展，县域经济均衡发展，中心镇培育成效明显，建成2000个左右的秀美乡村点。

——脱贫攻坚力度进一步加大。到2018年，现行标准下的23093个贫困人口全部脱贫，34个贫困村全部退出。到2020年，力争使我市农村最低生活保障标准超过国家贫困标准的40%，贫困村基本公共服务主要领域指标接近全市平均水平。

——群众幸福指数进一步提升。民生支出占一般公共预算支出的比重达75%，城乡居民人均可支配收入分别年均增长9%左右，城乡基本养老保险和医疗保险参保率分别达95%、98%以上，义务教育巩固率达到99%以上。

——生态环境优势进一步凸显。单位GDP能耗年均下降2.7%，主要污染物排放量控制在省政府下达的指标范围内，主要河流监测断面100%稳定达到或优于Ⅲ类水质，空气质量优良率稳定在90%以上，森林覆盖率稳定在58.2%左右。

做好今后五年经济社会发展工作，实现上述目标，必须把握以下方面：

一是坚持“全面小康”这个主线。同步全面建成小康社会，强调的不仅是“小康”，更重要的是“全面”。我们要紧扣同步全面建成小康社会这一主线，千方百计在调整产业结构、完善基础设施、落实生态保护、缩小城乡差距、健全社会保障体系、提升公共服务水平上下功夫、补短板，推动鹰潭在更高起点、更高标准上实现全面小康人口全覆盖、区域全覆盖、领域全覆盖。

二是坚持“全域一体”这个导向。鹰潭在全域统筹方面具有得天独厚的优势。必须牢固树立同心同向发展意识和全市“一盘棋”思想，在产业空间布局、新型城镇化、公共服务供给等方面突出全域一体化发展理念，突破行政区划，立足全域谋划，在资源共享中聚优势，在整体联动中凝合力，推动鹰潭在全省全国大发展格局中赢得应有的位置。

三是坚持“四化同步”这个路径。协同推进新型工业化、信息化、城镇化、农业现代化，必须坚持走一二三产联动发展之路，走信息化与工业化深度融合之路，走工业化与城镇化良性互动之路，走城镇化和农业现代化相互协调之路，通过“四化同步”，进一步优化经济结构、产业结构，提升发展质量和效益，抢占我们未来发展的主动权和制高点。

四是坚持改革创新这个动力。唯改革者进，唯创新者强，唯改革创新者胜。在新一轮大发展面前，我们必须坚持向改革开放要动力，向创新创业要活力，向特色优势要竞争力，优化提升开放平台，健全完善市场体系，推动政府职能转变，理顺体制机制，破除发展障碍，进一步累积后发优势，培育经济发展新动能，为建设“四个鹰潭”提供强劲动力。

三、今后五年工作的主要任务

围绕上述总体要求和奋斗目标，今后五年重点抓好以下六个方面的工作：

（一）聚力转型融合升级，构建现代产业新体系。深度对接“中国制造2025”、“互联网+”行动计划，按照创新引领、龙头带动、完善链条、集群发展的思路，以新型工业化为核心，协同推进现代服务业和现代农业加快发展，促进产业结构向中高端迈进。

1.深入实施工业强市战略。推进绿色世界铜都建设。不断完善“七位一体”铜产业体系建设，打造新型铜材产学研一体化基地、铜基新材料创新孵化器、铜文化创意产业园等新载体，繁荣铜贸易市场，做旺铜拆解加工区，加大铜产业科研和重点企业绿色技改投入，力争铜行业能效水效及资源综合利用率达到全国先进水平，铜产业实现主营业务收入突破5000亿元。打造千亿级非铜特色产业集群。加快推进以绿色水工、机械装备为主导的智能制造业，积极导入智慧水务、无人机、可穿戴设备、物联网、智能交通硬件、智能家居以及相关云配套服务，逐步向“智造鹰潭”转变。加快推进以节能照明和新能源为核心的光电新能源产业，着力培育智能化远程控制、智慧节能、新一代太阳能光伏等产品。加快推进以绿色食品和医药为重点的大健康产业，基本建成集中医医疗、养生保健、康复养老、健康旅游为一体的中医药健康服务体系。做大做强雕刻、眼镜、微型元件等特色产业基地。建设现代工业新城。加快沿320国道产业集聚升级带建设，构建“东部绿色铜都、中部高新服务、西部健康创意”三大产业集聚区，形成错位发展、布局合理、整体协调的市域产业格局。夯实园区平台，着力把鹰潭高新区打造成国家生态工业示范园区，加快贵溪经开区创建国家经济技术开发区步伐，支持余江工业园区扩区调区，力争全市工业园区拓园10平方公里以上，主营业务收入50亿元以上的企业达30家，市属工业园区主营业务收入达3000亿元。促进产城融合发展，推进园区和城镇基础设施、产业发展、市场体系、基本公共服务和社会管理、生态环保一体化建设，实现由功能单一的工业园区向多元布局的工业新城转变。

2.大力发展现代服务业。打响“龙虎天下绝”旅游品牌。围绕建设华东地区重要旅游节点城市，大力实施旅游产品多元化、旅游体验智慧化、旅游服务标准化、旅游合作区域化四大工程，进一步强化景区市场化运作功能，推动旅游产业园区建设，加大旅游产品宣传营销力度，不断增强龙虎山景区的市场竞争力和品牌影响力。围绕建设国家全域旅游示范区和旅游业改革创新先行区，壮大南北中三大旅游产业集群，大力发展休闲游、乡村游、自驾游、森林游等旅游新业态，促进旅游业全区域、全要素、全产业链发展，着力形成以龙虎山景区为龙头，以贵溪市、余江县为两翼，城景互动、城乡互补的大旅游框架。围绕建设“中华道都”，按照“中心城区做足元素、龙虎山景区做浓氛围、全市呼应发展”的布局，建设一批道文化标志性建筑，打造一批道文化产业基地，形成一批道文化产品。力争全市旅游总收入、旅游接待人次分别年均增长30%、25%以上。加快区域物流中心城市建设。健全现代物流体系，发展制造业物流、城乡商贸物流、口岸物流、电子商务物流等四大重点领域，形成“三组团、多园区”(6)的物流发展格局。完善市现代物流园、鹰西商贸园、国际物流中心功能，提升繁荣一批商贸物流专业市场，建成第四方物流(7)信息平台、公铁联运基地、公路港、快递产业园等一批重点项目，打造全方位、立体式现代物流体系。积极发展电子信息产业。坚持创新驱动、应用引领、项目支撑、产业集聚，建设电子信息产业园区，发展云计算、大数据、物联网等新一代信息技术、产业和应用，打造有特色的电子信息产业基地。提升现代金融业。加大股份制商业银行的引进力度，引导金融机构进行组织创新、产品创新和服务创新，大力引进天使投资、创业投资、风险投资等投资基金，加快企业上市步伐，不断提升直接融资占社会融资的比重。培育文化创意产业。深度挖掘拓展铜、黄蜡石、角山遗址、鬼谷子洞等文化内涵，建设一批文化休闲娱乐精品示范街和文化创意产业特色市场，逐步形成专业分工清晰、集群效益明显、功能配套完整的文化创意产业集群发展格局。

3.加快推进现代农业。大力实施藏粮于地、藏粮于技战略，落实最严格的耕地保护制度，建成高标准农田60万亩，确保基本农田面积稳定在113.15万亩以上，粮食总产量稳定在65万吨以上。大力发展规模农业，重点打造白鹤湖、雷溪、杨溪三个现代农业示范园区核心区，整合涉农资金，完善园区基础设施，积极引导新型农业主体向园区集中、先进农业生产要素向园区集聚。大力发展特色农业，加快农业结构调整，推进林下经济“12233”工程(8)建设，做精做强八大特色农业产业(9)，打造十个特色农业生产基地(10)，到2021年农产品加工业产值占农业总产值40%以上。大力发展品牌农业，精准扶持一批农业龙头企业，培育发展种养大户、家庭农场、农民合作社等新型经营主体，实施绿色生态农业十大行动(11)，着力打造一批以“道教龙虎山、绿色农产品”为主题的知名品牌。大力发展智慧农业，深入对接“123+N”工程(12)，力争农村电商覆盖全市所有行政村，现代农业示范园区核心区基本实现物联网应用。

（二）加速新型城镇化进程，形成城乡统筹新格局。坚持全域发展理念，全力推进国家新型城镇化综合试点城市建设，积极争创国家文明城市和国家卫生城市，加快形成布局合理、功能互补、智慧共享、城乡一体的城镇发展格局，走出具有鹰潭特色的新型城镇化道路。

1.全面实施“多规合一”。围绕省级“多规合一”试点建设，全力实施市城乡总体规划暨“多规合一”（2015—2030年），建立全市统一的“多规合一”信息管理平台，开展项目生成和业务审批协同作业，实现“一本规划管全域，一张蓝图干到底”。按照“一核两带四区”(13)城市发展布局，优化区域快速通道，建成余信贵大桥和余信贵大道，推进全域公共交通、公共服务设施一体化建设，加快贵溪西进、余江东拓和月湖新城建设步伐，形成全域一体、同城发展格局。

2.增强中心城区带动作用。繁荣发展信江新区，以中央商务区、高铁经济圈“双核”驱动为主导，推进信江北区路网等基础设施建设，完善文教卫等公共服务功能，大力发展金融商贸、旅游休闲、健康养老等产业，促进城市要素向信江新区集聚，着力把信江新区打造成为宜居宜业的核心城区。推进老城区提升改造，基本完成城镇棚户区和城中村改造，加快推进海绵城市建设，完善市区人行道升级改造、中心城区停车场等一批市政基础设施，贯通提升一批休闲街区、城市绿道、骑行公园和慢行系统，打造市民15分钟“健身圈”。推进城市服务网格化、精细化、数字化，及时整治影响市民生活的“城市病”，让城市生活更方便、更舒心、更美好。

3.打造国家新型智慧城市。全面实施国家信息惠民和国家智慧城市双试点工程，加快推进基础设施、应用服务和产业发展。积极采用云计算、大数据、物联网等新一代信息技术，不断提升城市光网能力，实现4G全覆盖、村村通宽带，构建先进的信息通信基础设施。大力推动“互联网+政务服务”，加快实施社会保障一卡通工程，率先建成一体化网上政务服务平台，实现“一号申请、一窗受理、一网通办”。落实“互联网+”行动计划，加速互联网与铜产业、物流业、旅游业、特色农业等产业深度融合，实现供需精准对接，提升综合效益。

4.加快城乡一体化发展。把握新型城镇化发展的战略机遇期，通过放宽户籍政策、提高医疗教育文化养老等公共服务水平、鼓励创新创业、引进大型企业和高等院校等举措，进一步做旺城市人气。以上清镇入选第一批中国特色小镇为契机，推进基础设施和公共服务设施建设，打造一批旅游、商贸、烘焙等特色小镇，进一步提升中心镇集聚能力。加速国道、省道升级改造，新改建农村公路2700公里，完善农村公路管理养护体系，加快镇村公交发展，全面推进“三安全一保障”(14)水利体系建设，推动秀美乡村建设向纵深发展。

（三）强化发展动力支撑，打造提速发展新引擎。坚持把改革推动、开放促动、项目带动、创新驱动作为加快发展的强力支撑，为转型升级、提速发展注入新动力、激发新活力、拓展新空间。

1.推进重点领域改革。加大供给侧结构性改革力度，认真落实“去产能、去库存、去杠杆、降成本、补短板”五大重点任务，提高全要素生产率，努力培育和形成新供给、新动力。持续推进“放管服”改革，深化商事制度改革，放开中介服务市场，建立完善中介服务平台。全面完成公车改革，加快事业单位分类改革。稳步实施全口径政府预算管理等财税体制改革，落实清费降价减税政策，推进财政资金统筹、安全、高效使用。整合公共资源交易平台，完成不动产登记制度改革。积极推进余江国家宅基地制度改革、农村改革试验区等重点改革和贵溪全国农村土地承包经营权抵押贷款试点，努力在全省乃至全国形成可复制、可推广的经验做法。实施供销合作社综合改革。深化全国综合行政执法改革试点，推进重点领域综合执法，实施行政检查“双随机”工作制度(15)。

2.推进全方位开放。深化区域协作，策应国家“一带一路”、赣南等原中央苏区振兴发展和赣东北扩大开放合作战略，加强与南昌都市圈、长江中游城市群和沪昆高铁经济带在市场要素整合、产业分工协作、公共信息共享等领域合作，努力打造长江经济带南翼重要战略支点。大力培育开放主体，创新承接产业转移模式及服务方式，积极承接东部地区加工贸易产业转移，实施招大引强攻坚计划和“央企入鹰、鹰商回归”提升工程，开展专业化招商、全端产业链招商和代理招商，着力引进一批行业领军企业、科技先导企业，力争全市引进省外2000万元以上项目资金、实际利用外资分别年均增长15%和9%。积极争取设立鹰潭公用性保税仓库，全面推进省口岸“单一窗口”试点建设。大力开拓新兴市场，建立健全外贸互联互通机制，力争外贸出口累计突破50亿美元。

3.推进项目带动战略。五年计划实施大中型建设项目544个、总投资3647亿元，加快推进信江航道渠化、鹰梅铁路、鹰潭机场、城市有轨电车等一批关系鹰潭长远发展的重大项目，力争建成花桥水利枢纽、贵溪—资溪高速公路、皖赣线电气化改造等项目。深化投融资体制改革，完善国有资产管理体制和国有资本投资运营机制，加快设立政府产业引导基金和高新技术产业发展基金，推广政府和社会合作项目PPP模式(16)，形成渠道畅通、成分多元、成本更低的项目融资机制。精准对接国家、省重点投资领域，积极争取国家政策、项目、资金支持。

4.推进全民创新创业。策应全省创新驱动“5511”工程(17)，积极开展与知名高校和科研院所合作，增强铜加工、生物工程、LED、先进制造业等领域协同创新力度，实施100项以上重大科技项目和重点创新项目，支持企业建设企业技术中心和重点工程实验室，力争市级以上科技创新平台达到100家以上。发展电子商务生态体系，制定电商发展中长期规划，构建电子商务公共服务平台，培育一批骨干企业和领军人物，做大做强电商产业集聚区，争创国家级电商进农村示范县（市），力争全市电子商务年交易额突破1000亿元。加快构建众创、众包、众扶、众筹等大众创业万众创新支撑平台，推进创新创业资助工程、大学生创业引领计划，培育“互联网+”新业态新模式。实施人才强市战略，制定人才行动计划，采取“送出去”与“引进来”相结合的方式，完善政府津贴、人才公寓和子女就学等优惠政策，采用校企合作和异地委培等模式，培养一批创新型人才和“鹰潭名师工匠”。积极推进质量兴市战略。

（四）推进生态文明建设，构筑绿色宜居新家园。围绕打造全省城乡最干净的城市，积极融入国家生态文明试验区建设，把生态文明建设贯穿经济社会发展各方面和全过程，加快建设天蓝、地绿、水净的家园，努力形成美丽江西“鹰潭样板”。

1.加强环境治理。开展大气污染综合防治，实施工业废气、建筑扬尘、机动车尾气专项治理，进一步提升环境空气质量。开展水污染综合防治，加大以信江及其支流为重点的中小河流与水库水质综合治理力度，中型水库和列入饮用水源的小型水库均达到II类水标准。抓好污水处理设施建设和运营，实现工业污水全面达标排放、城镇生活污水处理设施全覆盖和稳定运行、农村污水处理设施逐步完善。开展土壤污染综合防治，加强农业面源和规模化畜禽养殖污染防治，推进国家城乡生活垃圾第三方治理试点建设，建成生活垃圾焚烧发电厂二期，实现城乡生活垃圾全域无害化处理。

2.加强生态保护。实施新一轮造林绿化“六大工程”(18)，加强阳际峰国家自然保护区等重点区域管理，扩展森林生态功能区域。加强信江国家湿地公园和白鹤湖、白塔河等湿地公园保护力度，增强湿地生态系统能力。强化水生态保护，全面落实“河长制”，大力开展“清河行动”，加强饮用水源保护区等重点区域保护。深入推进国家工业绿色转型发展试点城市建设，突出做好链条延伸及产业协同、绿色改造及循环经济、绿色制造技术创新及产业化、构建绿色制造体系四大任务。推行绿色低碳生活方式，积极倡导居民绿色消费、绿色出行、绿色家居。

3.加强制度建设。实施严格的环境保护监管制度，制定生态环境保护地方性法规，将生态文明建设纳入法制化轨道。实施严格的污染物源头控制制度，落实新建项目环保准入机制和污染物排放许可制度。实施严格的土地资源管理制度，确保耕地占补平衡。完善绿色发展指标体系，建立健全领导干部任期生态文明建设责任制度、自然资源资产离任审计制度、保护发展森林资源目标责任制及生态环境损害责任终身追究制。

（五）倾力保障改善民生，共创幸福美好新生活。坚持民生优先、公平正义、共建共享，倾力办好一批民生实事，让改革发展成果更多、更实在地惠及广大人民群众，不断提升人民群众获得感和幸福感。

1.深入实施脱贫攻坚工程。做好保障扶贫，全面实施政策兜底，推进大病医疗救助、政府代缴养老保险和新农合等措施，有效衔接低保政策和扶贫政策，实现应保尽保。加强产业扶贫，推广“四位一体”产业扶贫模式(19)，支持贫困村“一村一品”特色扶贫产业发展，增强群众致富能力。开展安居扶贫，加强宜居贫困村基础设施建设和危房改造、村庄整治，到2017年基本解决建档立卡贫困户的安居问题，2020年建立覆盖全市农村的住房保障体系。实施智力和劳务扶贫，强化家庭贫困学生教育资助和职业培训，优先提供扶贫小额信贷支持创业扶贫，全面改善贫困地区义务教育薄弱学校基本办学条件。推进基础设施扶贫，加大财政扶贫投入，优先给予贫困村专项建设基金项目扶持，确保脱贫攻坚取得全面胜利。

2.加速城乡公共服务均等化。健全就业服务体系，推进千人创业万人就业工程，做好农村转移劳动力、城镇困难人员等重点群体就业工作，每年新增城镇就业1.96万人，城镇登记失业率控制在4.5%以内。健全社会保障体系，推行全民参保计划，强化以养老、医疗、失业、工伤、生育社会保险为重点的基本保障，全面实施统一的城乡居民基本养老保险制度，大力发展职业年金、企业年金、商业养老保险，鼓励发展补充医疗保险和商业健康保险。健全教育服务体系，深化教育领域综合改革，统筹城乡义务教育资源均衡配置，推进优质教育资源共享，完成教育信息化试点建设任务，加快普及学前教育和高中阶段教育，加强职业教育、特殊教育和老年教育，积极创建本科层次高等院校，打响“学在鹰潭”品牌。健全医药卫生服务体系，推进健康鹰潭建设，全面深化城市和县级公立医院综合改革，建成启用市妇幼保健新院、人民医院信江新院等一批专科医院，实现基本公共卫生服务均等化全覆盖。健全文化体育服务体系，积极实施文化精品、文化品牌、文化阵地、文化保护工程，建成启用“四馆”(20)项目，丰富群众精神生活。深入开展全民健身活动，大力发展健身休闲产业。健全养老服务体系，支持社会力量兴办养老服务机构，加快居家服务网点建设，基本建成以居家为基础、社区为依托、机构为补充，医养结合、功能完善、规模适中、覆盖城乡的养老服务体系。

3.增强公共安全保障水平。深化法治鹰潭建设，全面实施“七五”法治教育规划，形成全社会尊法学法守法用法自觉。加快建设以信息化为支撑的立体化社会治安防控体系，完善社会治安综合治理目标管理责任制，严密防范、依法惩治违法犯罪活动。推行“阳光信访”，引入第三方力量参与信访维稳和纠纷化解，实行网上受理办理，妥善化解矛盾纠纷。建成市级公共信用信息平台，着力打造“信用鹰潭”。实施食品安全战略，努力创建国家食品安全城市。坚持最严格安全生产制度，强化安全生产主体责任落实，坚决遏制重特大安全事故。强化防灾减灾指挥能力建设，健全突发公共事件应急处置体系，保障人民生命财产安全。

同时，深入推进军民融合发展，加强国防教育、人防民防、民兵预备役建设，进一步做好统计、档案方志、广播电视、民族宗教、外事侨务、涉台事务、气象、老龄和残疾人等工作，支持各民主党派、工商联、知识分子联谊会和工会、共青团、妇联等人民团体的工作。

（六）切实加强自身建设，塑造执政为民新形象。面对经济发展新常态，我们必须加快转变政府职能，加强创新型、服务型、法治型、廉洁型政府建设，为同步全面建成小康社会提供坚强保障。

1.开拓进取，建设创新型政府。始终把五大发展理念贯穿于经济社会发展各个环节，更加重视科学性研判、前瞻性考虑、可行性分析、系统性运作，善于向市场要资源、要活力、要手段。坚持把改革创新作为政府工作的永恒主题，以改革攻坚破除体制机制羁绊，在严峻挑战中抓住机遇，在激烈竞争中争取主动。推进学习型政府建设，增强互联网思维，加快提高网络管理水平，学网、懂网、用网，推进政府决策科学化、社会治理精准化、公共服务高效化，用信息化手段更好感知社会态势、畅通沟通渠道、辅助决策施政。

2.转变作风，建设服务型政府。强化进取意识、担当意识，把“马上就办、办就办好”作为基本准则，完善政府决策制定、工作推进、沟通协调三大机制，多做打基础、利长远、惠民生的实事好事。加快转变政府职能，精简办事程序，缩短办理时限，打通服务企业、联系群众的“最后一公里”。始终保持“一着不让抓落实”的紧迫感和责任感，加强对重大决策部署落实情况的督促检查，建立健全工作台账机制、绩效考核机制，真正把精力用在抓落实上、把功夫下在见实效上。

3.依法行政，建设法治型政府。严格依照法定权限和程序行使权力，自觉接受人大及其常委会法律监督，积极执行人大决议决定，坚持重大决策出台前向人大报告，认真办理人大代表的议案和建议。主动接受政协民主监督，认真办理政协委员提案，广泛听取各民主党派、工商联和无党派人士意见。健全政府信息发布制度，推进决策公开、执行公开、管理公开、服务公开、结果公开。健全政府法律顾问制度，完善重大事项依法决策机制、重大执法决定依法审查机制和政府立法机制，确保政府的一切工作在法律的框架下进行。

4.严守纪律，建设廉洁型政府。始终坚持不忘初心，把纪律和规矩挺在前面，认真履行党风廉政建设主体责任，强化“一岗双责”，完善惩治和预防腐败体系，推进反腐倡廉制度创新，加强行政监察和审计监督，坚决查处各类违法违纪案件。全面落实中央八项规定精神和国务院“约法三章”及省市各项规定，坚决抵制“四风”，厉行节约、反对浪费，严控“三公”经费支出。巩固拓展党的群众路线教育实践活动、“三严三实”专题教育和“两学一做”学习教育成果，推动作风建设常态化、长效化。

各位代表，新的目标激励人心，新的任务艰巨光荣，新的征程催人奋进。让我们紧密团结在以习近平同志为核心的党中央周围，在省委、省政府和市委的坚强领导下，紧紧依靠全市人民，坚定信心、鼓足干劲，开拓创新、扎实工作，为顺利完成“十三五”规划确定的各项目标任务，为建设富裕秀美宜居和谐鹰潭、同步全面建成小康社会而努力奋斗！
